# Organotypic slice culture based on in ovo electroporation for chicken embryonic central nervous system

**DOI:** 10.1111/jcmm.14080

**Published:** 2018-12-18

**Authors:** Ciqing Yang, Xiaoying Li, Shuanqing Li, Xuejun Chai, Lihong Guan, Liang Qiao, Han Li, Juntang Lin

**Affiliations:** ^1^ Stem Cells & Biotherapy Engineering Research Center of Henan College of Life Science and Technology, Xinxiang Medical University Xinxiang China; ^2^ Henan Key Laboratory of Medical Tissue Regeneration Xinxiang China; ^3^ Department of Anatomy Xi'an Medical University Xi'an China; ^4^ Advanced Medical and Dental Institute, University Sains Malaysia Bertam Penang Malaysia; ^5^ College of Biomedical Engineering, Xinxiang Medical University Xinxiang China

**Keywords:** central nervous system, chicken embryo, in ovo electroporation, optic tectum, organotypic slice culture, spinal cord

## Abstract

Organotypic slice culture is a living cell research technique which blends features of both in vivo and in vitro techniques. While organotypic brain slice culture techniques have been well established in rodents, there are few reports on the study of organotypic slice culture, especially of the central nervous system (CNS), in chicken embryos. We established a combined in ovo electroporation and organotypic slice culture method to study exogenous genes functions in the CNS during chicken embryo development. We performed in ovo electroporation in the spinal cord or optic tectum prior to slice culture. When embryonic development reached a specific stage, green fluorescent protein (GFP)‐positive embryos were selected and fluorescent expression sites were cut under stereo fluorescence microscopy. Selected tissues were embedded in 4% agar. Tissues were sectioned on a vibratory microtome and 300 μm thick sections were mounted on a membrane of millicell cell culture insert. The insert was placed in a 30‐mm culture dish and 1 ml of slice culture media was added. We show that during serum‐free medium culture, the slice loses its original structure and propensity to be strictly regulated, which are the characteristics of the CNS. However, after adding serum, the histological structure of cultured‐tissue slices was able to be well maintained and neuronal axons were significantly longer than that those of serum‐free medium cultured‐tissue slices. As the structure of a complete single neuron can be observed from a slice culture, this is a suitable way of studying single neuronal dynamics. As such, we present an effective method to study axon formation and migration of single neurons in vitro.

## 1 INTRODUCTION

Organotypic slice culture is an effective and well‐established method of maintaining tissue ex vivo. Tissues are able to maintain normal intercellular contact and differentiation patterns, as well as near‐normal physiological and morphological characteristics, thereby avoiding or reducing deficits between standard cell culture and animal modelling techniques. Organotypic slice culture techniques provide a good method for studying central nervous system (CNS) development, but the real breakthrough in this technology came from the roller‐tube technique pioneered by Gähwiler.^1^, ^2^, ^3^ Using this technique, slices derived from various brain regions have been kept in culture for up to several weeks. In 1991, Stoppini et  al used a sterile, transparent and porous membrane to culture the rat hippocampus, achieving good results, and thus establishing a simple method for organotypic culture of nervous tissue.^4^ Brain slice culture can successfully simulate the process of neural development similar to that observed in vivo, and provide experimental evidence for organ‐like brain slices, as an alternative to the in vivo brain.^5^ This method has been effectively applied to various fields of experimental neuroscience.^6^, ^7^, ^8^, ^9^

Today, organotypic brain slice culture is mainly applied to mouse and rat tissue, as well as for disease modelling, such as with transgenic mice.^10^, ^11^, ^12^, ^13^, ^14^, ^15^, ^16^, ^17^ However, there are few reports related to the study of organotypic slice culture using chicken embryos.^18^, ^19^ The chicken embryo is a good animal model, used for both developmental biology and neurobiology studies. Particularly for the study of embryonic CNS development, the chicken embryo has the advantages of being abundant, easy to manipulate and easy to access to collect material from. With the emergence of in ovo electroporation technology using the chicken embryo, the ectopic expression of exogenous genes can easily be achieved in the CNS. We thus developed an organotypic slice culture combined with in ovo electroporation technology to study the dynamic changes of neurons under exogenous gene expression during chicken CNS development. This method will provide a new strategy for the functional study of exogenous genes in the development of the chicken CNS.

## 2 MATERIALS AND METHODS

### 2.1 Culture media

The reagents used included slice culture free serum media (48 mL of Neurobasal (Thermo Fisher, USA), 1 mL of B‐27^®^ Serum‐Free Supplement (50X) (Thermo Fisher), 0.5 mL of 1% GlutaMAX (Thermo Fisher), 0.5 mL of 1% penicillin‐streptomycin (Sigma), 25% horse serum media (37.5 mL of free serum media, 12.5 ml horse serum [HyClone, USA]) and artificial cerebrospinal fluid (aCSF) (120 mmol/L NaCl, 3.5 mmol/L KCl, 1.3 mmol/L MgCl_2_, 2.5 mmol/L CaCl_2_, 1.25 mmol/L NaH_2_PO_4_, 25.6 mmol/L NaHCO_3_ and 10 mmol/L glucose).^20^

### 2.2 Embryo preparation

Fertilized eggs were obtained from a local farm and incubated (HWS‐150 Incubator, JingHong, China) at 37.8°C and 65% humidity. The Hamburger and Hamilton system was used to stage the embryos.^21^ Embryos were studied from stages 17 (E2.5) to 38 (E12), with at least five embryos collected from each stage.

### 2.3 Spinal cord in ovo electroporation and tissue section

**Figure 1:**
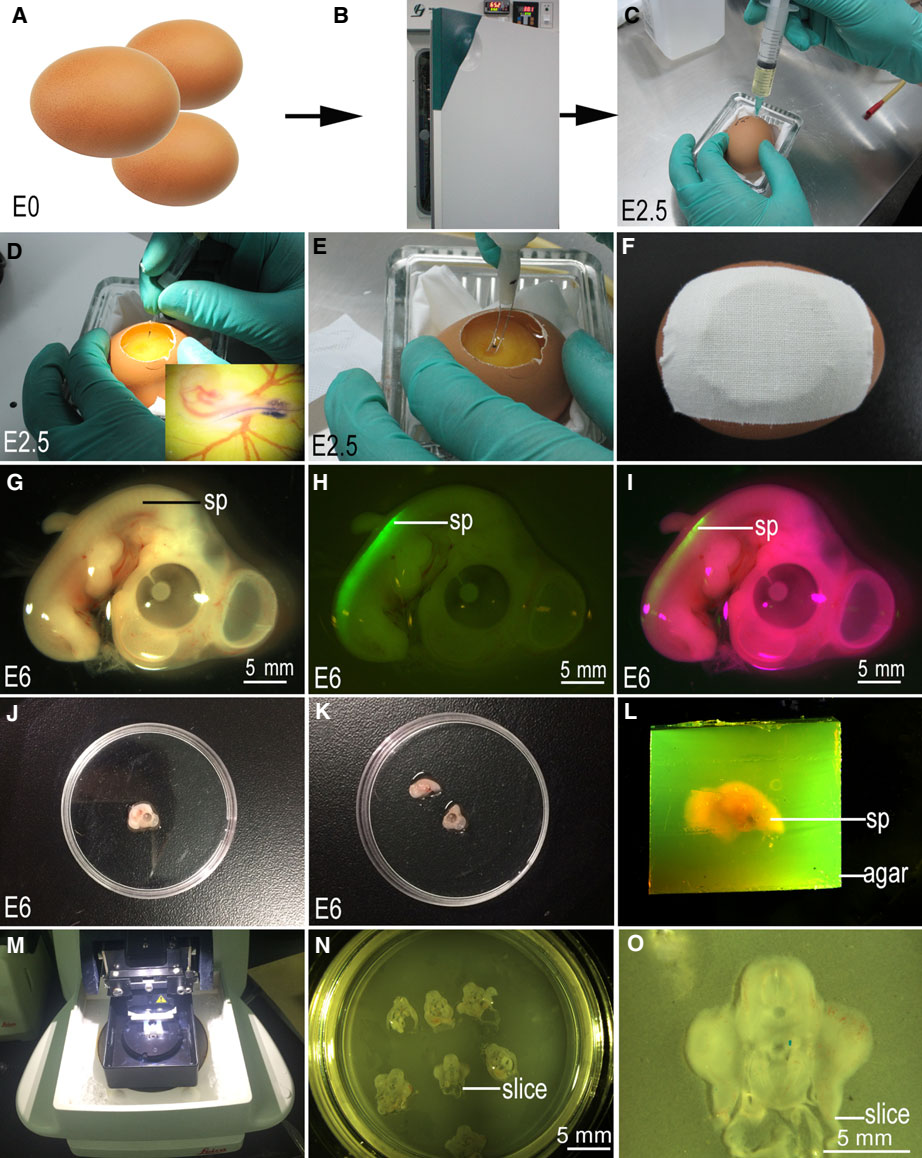
Spinal cord in ovo electroporation and tissue section. (A‐F,J‐M) were imaged using a canon camera; (G‐I,N‐O) were imaged using a stereo fluorescence microscope; (A,B) Incubation of fertilized eggs; (C) Removal of approximately 3‐4 mL of albumin; (D) Injection of plasmid into spinal cord; E: In ovo electroporation; (F) Incubation of the eggs; (G‐I) Material collection; (J‐K) Green fluorescent protein‐positive spinal cord collection; (L) Samples embedded in 4% agar; (M) Tissues sectioned; (N‐O) Sections mounted on the membrane of millicell cell culture insert; sp: spinal cord; Scale bar = 5 mm in (G‐I) and (N‐O)

We used a pCAGGS‐green fluorescent protein (GFP) plasmid to drive GFP expression as a cellular marker. All plasmids were purified using a plasmid extraction kit (Cwbio, China), as per the manufacturer's instructions, and diluted in water. The in ovo electroporation protocol used was modified from a previously published study and a stereomicroscope was used to assist with all steps of electroporation.^22^ Briefly, fertilized eggs were incubated until stage 17 (E2.5) (Figure 1A,B), after which 3‐4 mL of albumin was removed without disrupting the yolk (Figure 1C). The shell was cut carefully with a pair of curved scissors to construct a 1‐2 cm diameter window without touching the embryo. A mixture containing 0.25 µg/µL of pCAGGS‐GFP plasmid and Fast Green dye (0.01%) was injected and loaded into the spinal cord with a mouth pipette until the dye filled the entire space (Figure 1D). Electrodes were then immediately placed in parallel on either side of the embryonic neural tube (Figure 1E). A total of six 18‐volt pulses, lasting 60 ms and separated by a 100 ms pause, were emitted. After electroporation (CUY‐21 Electroporator, Nepa Gene, Japan), the electrodes were carefully removed and the egg was sealed with tape (Figure 1F). The eggs were then placed back into the incubator until they reach the desired stage (26 [E6]) for sample collection, sectioning and slice culture. For bromodeoxyuridine (BrdU) labelling, 5 μg/μL of BrdU was added into the embryo 24 hours prior to fixation and sample collection.

At stage 26 (E6), GFP‐positive embryos were selected (Figure 1G‐I) and fluorescent expression sites were cut under a stereo fluorescence microscope (Figure 1J,K). Selected tissues were embedded in 4% agar, which was pre‐cooled to 40°C (Figure 1L). The tissues were sectioned using a vibratory microtome (VT1200S, Leica, Germany) and the 300 μm thick sections were mounted on a membrane of millicell cell culture insert (PICM03050, Millipore) (Figure 1M‐O). In the process of tissue sectioning, aCSF was added to the buffer plate of the vibratory microtome in order to protect the cut tissue (Figure 1M‐O).

### 2.4 Optic tectum in ovo electroporation and tissue section

**Figure 2:**
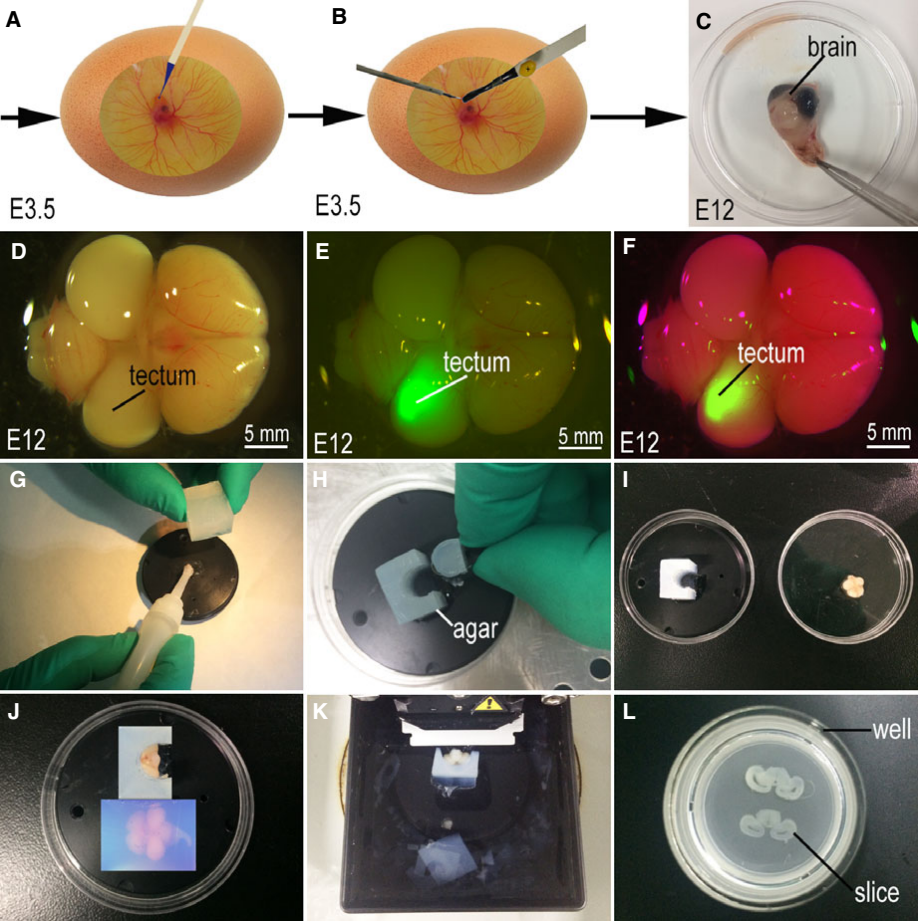
Optic tectum in ovo electroporation and tissue section. (A‐C,G‐L) were imaged using a canon camera; (D‐F) were imaged using a stereo fluorescence microscope; (A) Injection of plasmid into optic tectum; (B) In ovo electroporation; (C) Incubation of the eggs up to stage 38 (E12); (D‐F) Green fluorescent protein‐positive brain collection; (G) 4% agar block; (H‐I) Groove cutting on one side of agar block; (J) Samples embedded in 4% agar; (K) Tissues sectioned; (L) Sections mounted on the membrane of millicell cell culture insert; Scale bar = 5 mm in (D‐F)

The in ovo electroporation protocol was modified from a previous study.^23^ Briefly, fertilized eggs were incubated until stage 17 (E2.5), after which 6 mL of albumin was removed from the egg without disrupting the yolk.^23^ One day later (E3.5), the shell was cut carefully with a pair of curved scissors to construct a 1‐2 cm diameter window without touching the embryo. A mixture, containing 0.25 µg/µL of pCAGGS‐GFP plasmid and Fast Green dye (0.01%), was injected and loaded into one side of the optic tectum using a mouth pipette until the dye filled the entire space (Figure 2A). Electrodes were then immediately placed in an overlapping position on both sides of the embryonic optic tectum (Figure 2B). A total of six, 15‐volt pulses were administered, lasting 60 ms each and separated by a 100 ms pause (Figure 2B). After electroporation (CUY‐21 Electroporator, Nepa Gene), the electrodes were carefully removed and the egg was sealed with tape. The eggs were then placed back into the incubator until they reach the desired stage 38 (E12) for sample collection (Figure 2C), sectioning and slice culture.

At stage 38 (E12), GFP‐positive embryos were selected using a stereo fluorescence microscope (Figure 2D‐F). The optic tectum section was cut according to two different methods. Initially, the 4% pre‐cooled agar is cut into a square according to the size of the tissue (Figure 2G), and pasted to the tray of the vibratory microtome using a strong adhesive (Figure 2G). A groove was next cut into one side (Figure 2H‐I), with the optic tectum of the chicken brain towards the groove, and the sides of the olfactory bulb affixed to the tray (Figure 2J). Another method was to take selected tissues and embed the selected tissues in 4% agar that had been previously pre‐cooled to 40°C (Figure 2J). The tissues were coronally sectioned on a vibratory microtome (VT1200S, Leica) and 300 μm thick sections were mounted on a membrane of millicell cell culture insert (PICM03050, Millipore) (Figure 2K, L). In the process of tissue sectioning, aCSF was added to the buffer plate of the vibratory microtome in order to protect the cut tissue (Figure 2K‐L).

### 2.5 SLICE CULTURE

The Millicell cell culture insert well was placed in a 30 mm culture dish containing 1 ml of slice culture media. The dishes were transferred to an incubator at 37°C and 5% CO_2_. The slices were cultured for 24 h before media replacement, and then the media replenished once in every two days. Tissues were maintained for 7 days. The morphological structure of the cultured slice was analysed after 48 h by confocal microscopy (Olympus ix81, Japan). For BrdU labelling, 5 μg/μL of BrdU was added to the media 24 hours prior to sample collection.

### 2.6 TISSUE SECTION

Samples of the optic tectum and spinal cord tissue containing GFP were collected. Embryos of a sufficient size were fixed in 4% formaldehyde solution for 24 hours. Following the fixation, the tissue was immersed in an 18% sucrose solution, embedded in Tissue‐Tec OCT compound (Sakura Finetek, USA), frozen in liquid nitrogen and stored at −80°C until use. Samples were sectioned on a cryotome (Leica 1850, Germany) and the 20 μm thick sections were mounted on poly‐L‐lysine coated slides.

### 2.7 Immunohistochemistry

For immunohistochemistry, sections were fixed with 4% paraformaldehyde in PBS for 15 minutes at 37°C. Following another TBS wash, a blocking solution (2% sheep serum, 4% bovine serum albumin, 0.3% Triton X‐100% and 0.1% sodium azide in Tris‐buffered saline (TBS, sheep serum and bovine serum albumin; Beijing Dingguo co. LTD, China) was applied to tissue sections for 1 hour at room temperature. Primary antibodies were then applied overnight at 4°C. Primary antibodies used in this study included rabbit anti‐caspase‐3 polyclonal antibody (Proteintech, China, 1:100 dilution), mouse anti‐BrdU monoclonal antibody (ZSGB‐BIO, China; 1:100 dilution), rabbit anti‐Map2 polyclonal antibody (Abcam, 1:300 dilution), mouse anti‐NeuN monoclonal antibody (Abcam, 1:300 dilution), mouse anti‐neurofilament (NF) (1:10 dilution), rabbit anti‐glial fibrillary acidic protein (GFAP) polyclonal antibody (Abcam, 1:300 dilution) and rabbit anti‐Iba1 monoclonal antibody (Abcam, 1:300 dilution). For BrdU detection, sections were incubated in 2 N HCl for 30 minutes followed by 0.1 mol/L Na_2_B_4_O_7_ (pH 8.5) and then rinsed several times in TBS before the incubation with anti‐BrdU. Next, the appropriate secondary antibodies, whether goat‐anti‐rabbit Cy3‐labelled (Jackson Immuno Research, Europe Ltd, 1:1000 dilution), goat‐anti‐mouse Cy3‐labeled (Jackson Immuno Research, Europe Ltd, 1:1000 dilution) or goat‐anti‐rabbit FITC‐labelled (ZSGB‐BIO, China; 1:100 dilution) secondary antibodies were applied for 2 hours at 25°C. A similar process was employed for double staining. Finally, DAPI (4ʹ,6‐diamidino‐2‐phenylindole, DAPI, Roche, Germany) was used to stain all cell nuclei.

### 2.8 Microscopy

The whole embryo or brain was imaged under a stereo fluorescence microscope (M205FA, Leica, Germany) equipped with a digital camera (DFC425C, Leica, Germany). Tissue sections were imaged by confocal microscopy (Olympus ix81). Digitized images were adjusted for contrast and brightness using Photoshop software (Adobe, USA).

### 2.9 Time‐lapse imaging

For time‐lapse imaging, we set up a climate chamber on an inverted confocal microscope platform. The temperature of the climate chamber was 37°C and contained 5% of carbon dioxide. In order to clearly observe the morphology of neurons, the confocal 40× long focus lens was used. A culture slice with complete morphology and GFP expression was selected from a culture dish under an inverted fluorescence microscope. The dish containing the membrane insert with the slice and containing 1 mL of slice culture medium was placed into the climate chamber of the confocal microscope. The resolution was set to 800 by 1000 pixels and the time‐lapse series was initiated at a rate of one z‐stack every 10 minutes. Time‐lapse series were analysed using Photoshop software (Adobe).

## 3 RESULTS

### 3.1 Combination of in ovo electroporation and slice culture for the study of gene function

**Figure 3:**
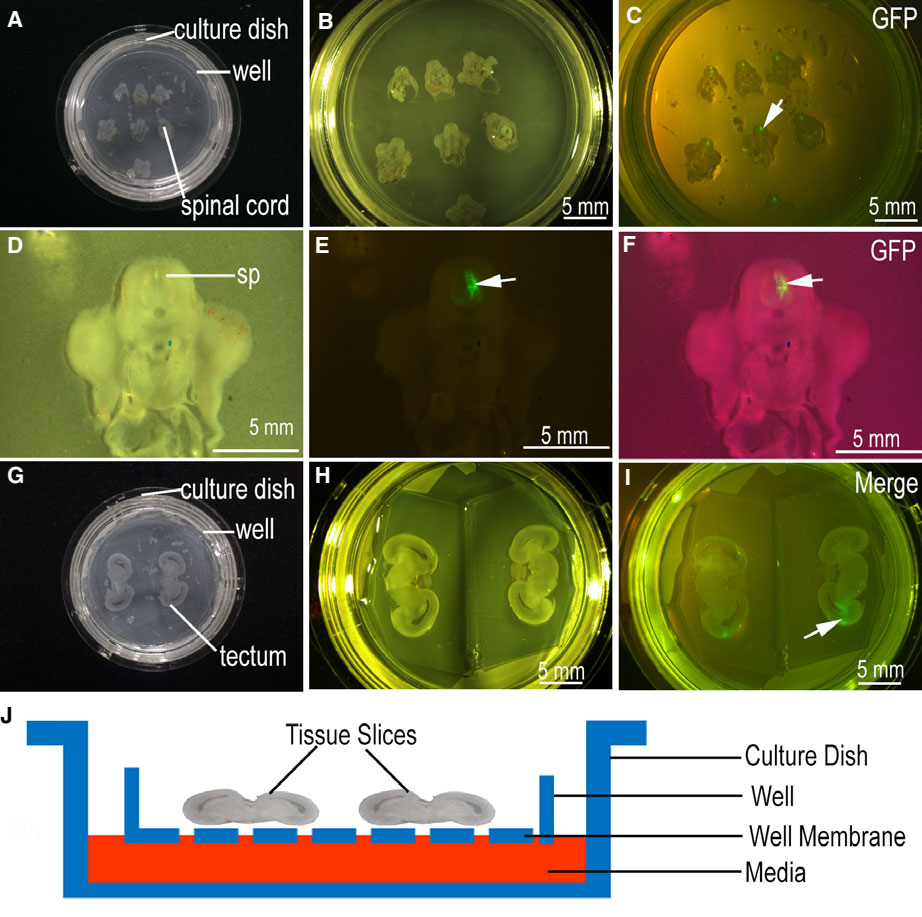
Slice culture. (A,G) were imaged using a canon camera; (B‐F,H‐I) were imaged using a stereo fluorescence microscope; (A‐F) Spinal cord slices culturing; (G‐I) Optic tectum slice culturing; (J) Tissue slice culture model. Scale bar = 5 mm in (B‐C,E‐F,H‐I)

We combined in ovo electroporation and slice culture technology to study gene functions in the spinal cord and optic tectum. With regard to the spinal cord, in ovo electroporation was conducted at stage 17 (E2.5). The samples were sectioned at stage 26 (E6), and GPF‐positive slices were selected for culture using a stereo fluorescence microscope (Figure 3A‐F). In general, in the process of spinal cord section culture, six to eight slices can be placed in one well membrane (Figure 3A). When positioning slices, it is important to avoid any contact between slices by leaving enough space between them and to select only the GFP‐positive tissue slices (Figure 3B,C). In order to ensure the quality of results, the slices should not fold or break and the expression of GFP should be accurate in one side of the spinal cord (Figure 3D‐F). With regard to tectum slice culture, two slices can be placed in one well membrane (Figure 3G). As the migration of transfected neurons in the ventricular zone is what mainly observed in the optic tectum, GFP expression should be restricted to the ventricular zone (Figure 3H‐I). The tissue slices were placed in full contact with the medium and exposed to the air so as to prevent tissue necrosis (Figure 3J).

### 3.2 Serum‐free medium cultured slice neurons lose strict regulation

**Figure 4:**
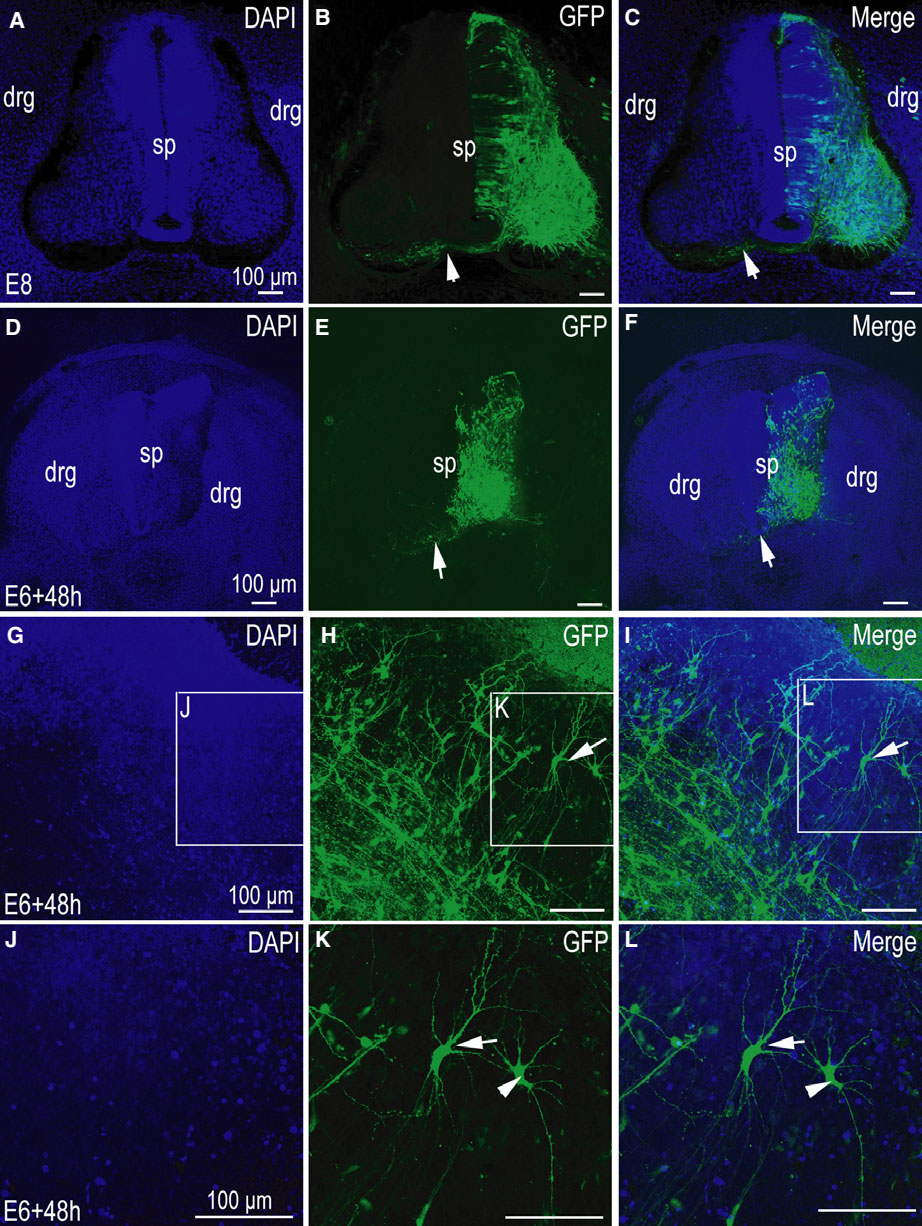
Differences in tissue morphology and neuronal structure between cultured slices and tissue sections in the spinal cord. (A‐P) were imaged using a confocal microscope. (A‐C) Control group pCAGGS‐green fluorescent protein (GFP)‐positive section at stage 26 (E6). (A) 4ʹ,6‐diamidino‐2‐phenylindole (DAPI) nuclear staining, (B,C) GFP expression and merged images. (D‐F) pCAGGS‐GFP‐positive slices at stage 26 (E6) cultured for 48 h; (D) DAPI nuclear stain; (E,F) GFP expression and merged images; (G‐L) Single neuron in culture slices of spinal cord, DAPI nuclear stain (G, higher magnification in J), GFP expression (H, higher magnification in K), and merged image (I, higher magnification in L). sp, spinal cord; drg, dorsal root ganglion. Arrows (→) in (B‐C,E‐F) denote commissural axons. Scale bars, 100 µm in (A,D,G,J) for (A‐L) respectively

In tissue sections, patterns of migration of GFP‐positive neurons tend to be regular and axonal projections are strictly regulated by various factors in the body (Figure 4A‐C). We were able to observe transfected commissural axons on one side of the spinal cord projecting to the contralateral side through the floor plate (Figure 4B, C). Following 48 hours of slice culture, while GFP‐positive neurons were still be seen on one side of the spinal cord, the direction of neuronal migration and projection of commissural axons were altered (Figure 4E,F).

### 3.3 Serum‐free medium culture slices lose the original structure of the CNS

**Figure 5:**
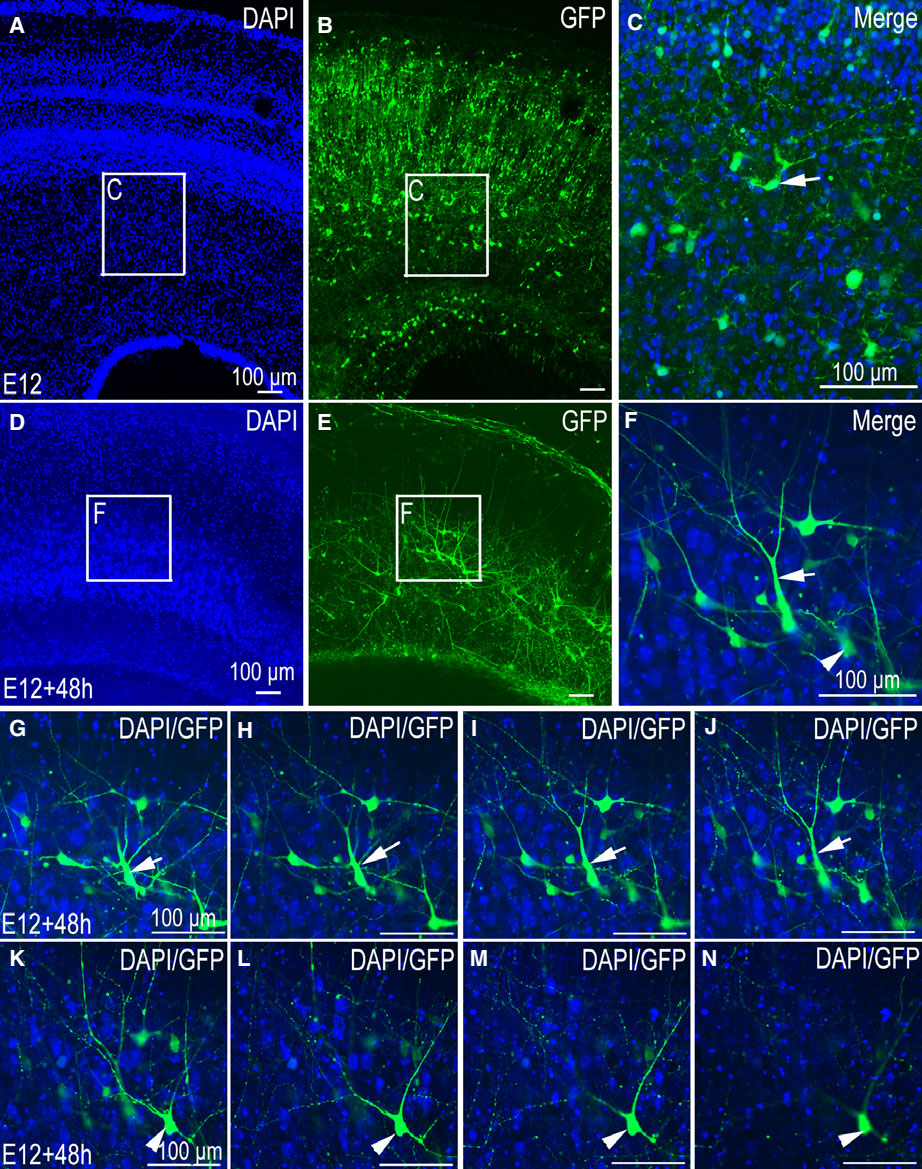
Differences in tissue morphology and neuronal structure between slice cultures and tissue sections in the optic tectum. (A‐N) were imaged using a confocal microscope. (A‐C) Control group, pCAGGS‐GFP‐positive section at stage 38 (E12). (A) 4ʹ,6‐diamidino‐2‐phenylindole (DAPI) nuclear staining; (B) GFP expression. Higher magnification merged images of (A) and (B) are shown in (C). (D‐F) pCAGGS‐green fluorescent protein (GFP)‐positive slices at stage 38 (E12) culture to 48 h; (D) DAPI nuclear staining; (E) GFP expression; Higher magnification merged images of (D) and (E) are shown in (F). (G‐N) A series image of (F) scanning different layers in culture slices of optic tectum. Arrows (→) in (C) denote single neuron in section, Arrows (→) in (F‐N) denotes single neuron of different layers in culture slices. Scale bars, 100 µm in (A,C,D,F,G,K) for (A‐N) respectively

Compared with the tissue sections taken directly from the embryo (Figure 4A‐C), the most significant difference in the cultured tissue is the loss of the original morphological structure (Figure 4D‐F). Following 48 hours of slice culture, the structure of the dorsal root ganglion (drg) in the spinal cord was blurred, and the boundary between the spinal cord and the surrounding tissue was no longer obvious (Figure 4D). Similar results were seen on culturing the optic tectum slice (Figure 5A‐F). While the optic tectum had a distinct layer structure in the freshly cut tissue sections (Figure 5A‐C), the boundary became blurred and the structure of each layer of the optic tectum was no longer obvious following tissue culture (Figure 5D‐F).

### 3.4 Comparison of tissue morphology and neuronal structure between serum‐free medium and 25% horse serum medium‐added cultured slice

**Figure 6:**
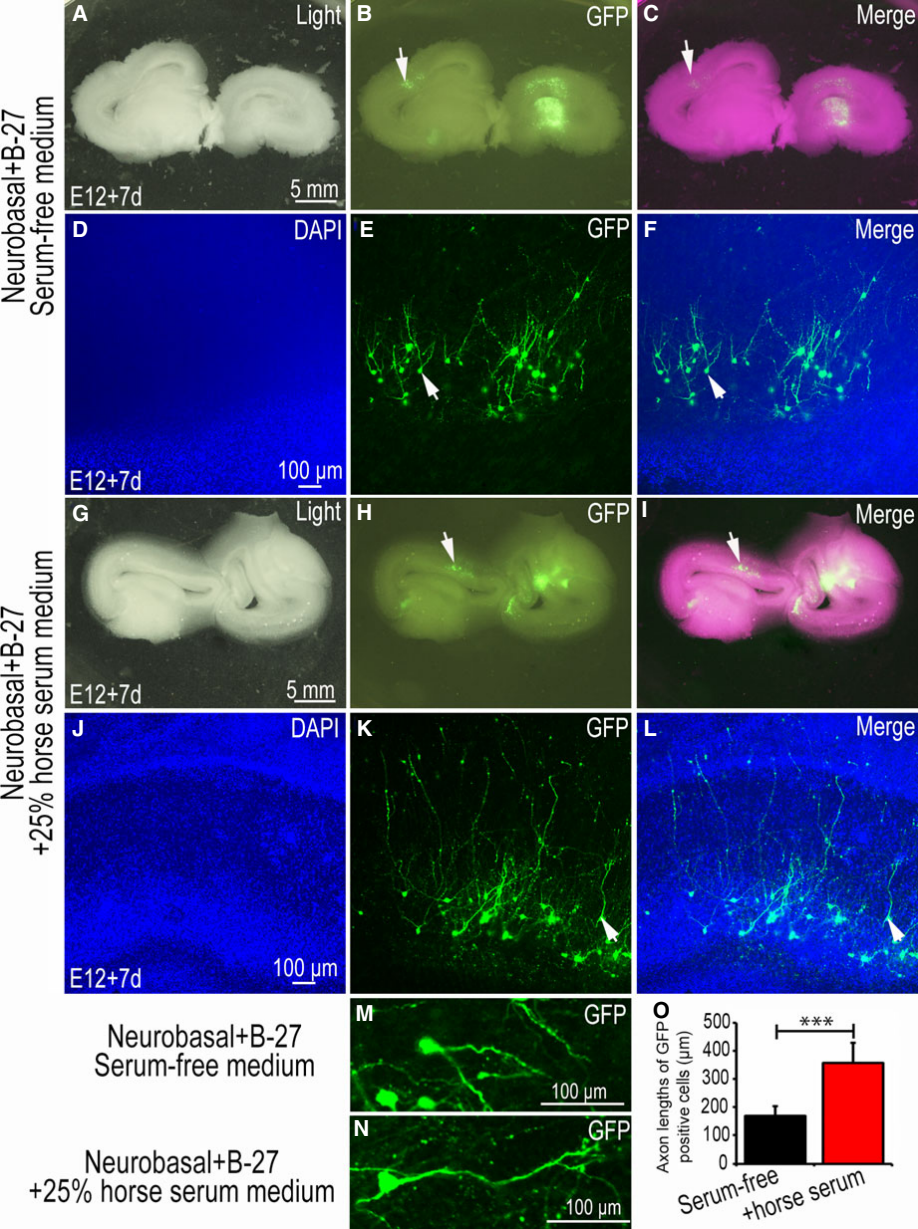
Comparison of tissue morphology and neuronal structure between serum‐free medium and 25% horse serum‐medium slice cultures. (A‐C) and (G‐I) were imaged using a stereo fluorescence microscope; (D‐F) and (J‐L) were imaged using a confocal microscope. (A‐F) used serum‐free medium (Neurobasal added B‐27 as the medium), (A‐C) cultured for 7 d, (D) 4ʹ,6‐diamidino‐2‐phenylindole (DAPI) nuclear staining; (E) Green fluorescent protein (GFP) expression, (D) and (E) merged images are shown (F). (G‐L) used 25% horse serum medium (Neurobasal added B‐27% and 25% horse serum as the medium), (G‐I) cultured for 7 d, (J) DAPI nuclear staining; (K) GFP expression, (J) and (K) merged images are shown in (L). Arrows (→) in (B‐C) and (H‐I) denote GFP‐positive areas in the tectum, Arrows (→) in (E‐F) and (K‐L) denote single neurons from GFP‐positive in culture slices from higher magnification images are show (M) and (N) respectively. (O) Axon lengths of GFP‐positive neurons were compared and the data presented as the mean ± SD. ***P < 0.001. Scale bars, 5 mm in (A,G) for (A‐C) and (G‐I), 100 µm in (D,J) for (D‐F) and (J‐L) respectively.

Over the course of the experiment, the tissue slices cultured in serum‐free medium lost their original morphology and structure, and patterns of neuronal migration also lost their strict regulation. Therefore, we added 25% horse serum in serum‐free medium for comparative purposes. We found that in serum‐free medium, the adhesive between the tissue slice and the insert culture dish membrane was very weak and would fall off easily on rinsing (Figure 6A‐C). In addition, the edge of the tissue was not smooth enough (Figure 6A). However, neurons transfected with GFP plasmids were clearly visible and distributed across different layers of the tectum (Figure 6B‐C). Further magnification (Figure 6B‐C arrow area) under confocal microscopy revealed that the layers of the optic tectum stained with DAPI were not distinctly stratified (Figure 6D). However, typical neurons can be clearly seen, suggesting that brain slices remain active in serum‐free medium, but do not guarantee good morphological structure (Figure 6E‐F). Tissue slices were similarly cultured in the medium containing 25% horse serum. It was found that the cultured tissue slices adhered strongly to the insert culture dish membrane were not desquamated by rinsing or even blowing slightly, and did maintain good morphology and structure (Figure 6G‐I). Greater magnifications revealed that the DAPI‐stained nuclei had a layered structure (Figure 6J), but that the layered structure was still different from that of the same time layer in vivo (Figure 5A). Compared with serum‐free medium, the length of GFP‐labelled axons in 25% horse serum culture were significantly longer than those in serum‐free medium (Figure 6K‐L), especially in the enlarged structure of a single neuron (Figure 6M,N). To shed light on this, we analysed the length of neuronal axons. Results showed that the length of axons in the medium containing 25% horse serum was 358.78 ± 70.48, which was significantly (*P* < 0.001) larger than that in the medium without serum, while the length of axons in the serum‐free medium was 170.06 ± 33.60 (Figure 6O).

### 3.5 Slice culture is suitable for the study of a single neuron dynamics

The analysis of single neurons in cultured‐tissue reveals an integral neuronal structure, including a very obvious dendrite (Figures 4G‐L and 5F). A complete single neuron can be observed by a scanning confocal microscope (Figure 5G‐N). Usually, as neurons have three‐dimensional spatial structure, this complete structure is difficult to observe in tissue sections (Figure 5C). Indeed, during the process of sectioning, parts of the neurites are usually lost (Figure 5C). Thus, in the observation process, incomplete structures are often acquired (Figure 5C). During slice culture, neural precursor cells can form neurons, and the protruding axons and the structure of the dendrites can be completely retained (Figure 5F). Despite the thickness of the cultured tissue slice, it is not possible to observe complete neuronal structures at low magnifications. However, the different layer structures of a single neuron can be observed by scanning confocal microscopy (Figure 5G‐N). In addition, in slice culture, a neuron's dynamic changes can be observed under the microscope. Combined with in ovo electroporation technology, we can study neuronal migration and axon formation following ectopic expression of exogenous genes (Figure 6A‐L).

**Figure 7:**
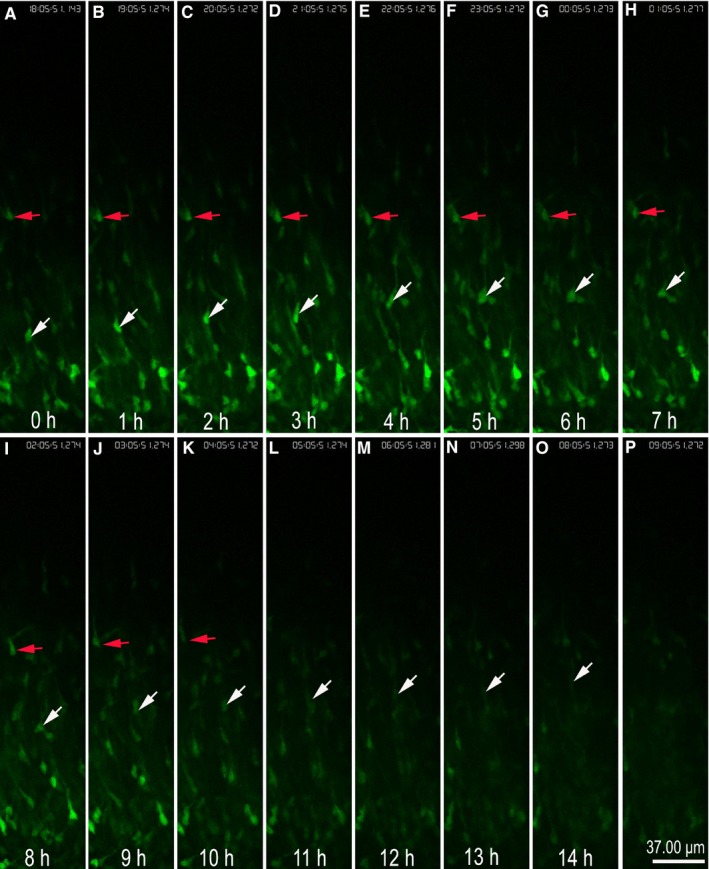
Time‐lapse showing the dynamic migration of neurons. (A‐P) were imaged using a confocal microscope. (A‐P) the time‐lapse result shows the process of dynamic migration of green fluorescent protein‐positive neurons within 0‐16 h. Red arrow (→) in (A‐K) denotes a same single neuron in section at different times as a control. White arrow (→) in (A‐O) denotes the dynamic change of a same single neuron in section at different times. Scale bars, 37.0 µm in (P) for (A‐P) (see Video S1)

In addition, during slice culture, using in ovo electroporation combined with time‐lapse technology, we can observe the dynamic migration of neurons. In this experiment, we collected time‐lapse data from the cerebral cortex of E14.5 mice over the course of 16 hours (Figure 7 A‐P). Following in ovo electroporation, the expression of reporter gene GFP will spontaneously fluorescence green allowing for the transfected neurons to be clearly observed by fluorescence microscopy. Over the course of slice culturing, live neuron conditions were simulated in vivo, causing neurons to mirror in vivo migratory patterns. Using confocal microscopy's time‐lapse function, parameters were set and scans obtained every 10 minutes. Following 16 hours of culture, we were able to trace the complete migration of single neurons (Figure 7 A‐P, see Video S1). Compared with the control cells (Figure 7, red arrow), the neurons (Figure 7, white arrow) observed in the time‐lapse images were uniformly moving outwards. This method can also be used to study the gain and loss of function of exogenous genes over the course of neuronal migration, especially in the context of dynamic changes which are unable to be observed in vivo.

### 3.6 Effects of in ovo electroporation on cell apoptosis and proliferation in slice cultures

**Figure 8:**
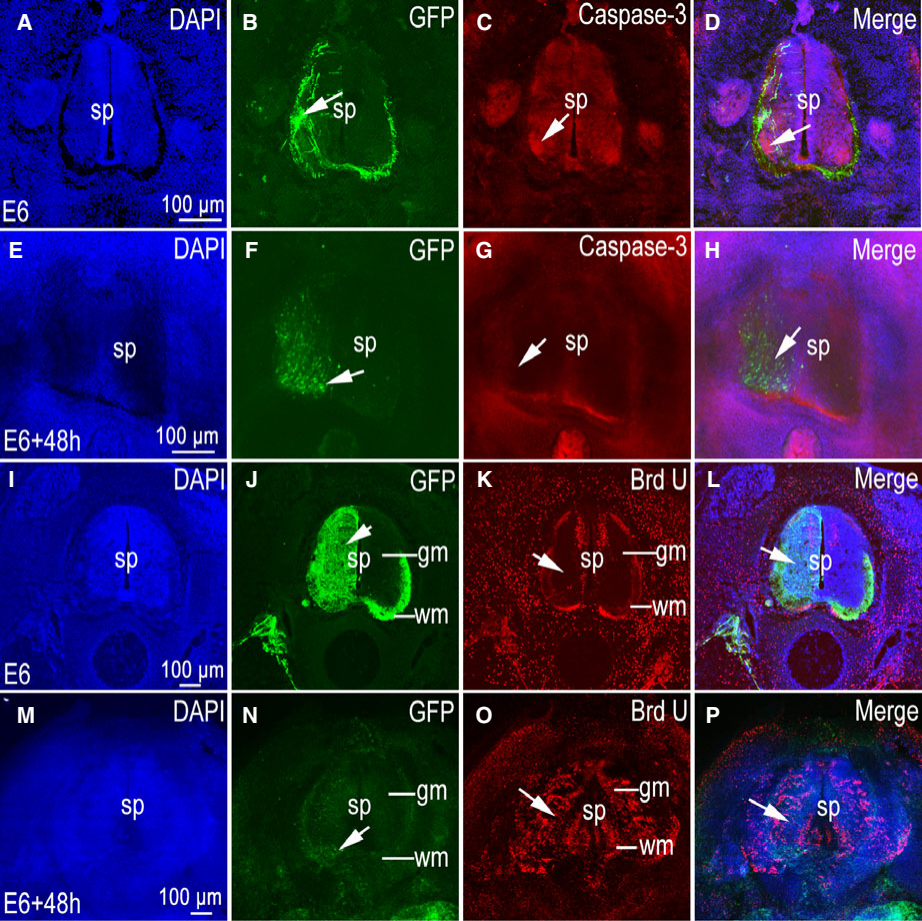
Effects of in ovo electroporation on cell apoptosis and proliferation in slice culture process. (A‐P) were imaged using a confocal microscope. (A‐C) Control group pCAGGS‐green fluorescent protein (GFP)‐positive section at stage 26 (E6), (A) 4ʹ,6‐diamidino‐2‐phenylindole (DAPI) nuclear staining (Blue); (B) GFP expression (green); (C) caspase‐3 expression (red); (D) merged images; (E‐H) pCAGGS‐GFP positive slice at stage 26 (E6) culture to 48 h; (E) DAPI nuclear stain (Blue); (F) GFP expression (green); G: caspase‐3 expression (red); (H) merged images. (I‐L) Control group pCAGGS‐GFP positive section at stage 26 (E6); (I) DAPI nuclear staining (Blue), (J) GFP expression (green), (K) Brd U expression (red), (L) merged images. (M‐P) pCAGGS‐GFP positive slice at stage 26 (E6) culture to 48 h; (M) DAPI nuclear stain (Blue); (N) GFP expression (green); (O) Brd U expression (red). Sp, spinal cord; gm, grey matter; wm, white matter. Arrows (→) in (B,F,G,N) denotes GFP‐positive area, in (C,G) denote caspase‐3 expression in GFP positive area, and in (K,O) denote Brd U expression in GFP positive area. Scale bars, 100 µm in (A,E,I,M) for (A‐P) respectively

In order to shed light on whether this technique will interfere with neuronal survival and apoptosis, we stained for caspase 3 and BrdU in both experimental and control groups. Results showed that caspase‐3 had low expression levels in both control group and slice culture tissue groups, and that its expression was not enhanced in the region of GFP‐positive regions (Figure 8A‐H). Results also indicated that neuronal transfection with GFP did not promote neuronal apoptosis in slice cultures. Between the control groups, BrdU staining revealed significant differences (Figure 8I‐P). No BrdU‐positive neurons were observed in grey matter areas of the control group (Figure 8K), while a large number of BrdU‐positive cells were observed in grey matter areas in the experimental group (Figure 8O). In control group and experimental groups, no difference in BrdU expression was seen between GFP‐positive side and contralateral sides of the spinal cord (Figure 8K,O). This indicates that the difference in the BrdU marker cells was not caused by the expression of GFP, but rather by the differences in the slice culture and the living tissues.

### 3.7 The expression of MAp2, NeuN and NF in cultured slices

**Figure 9:**
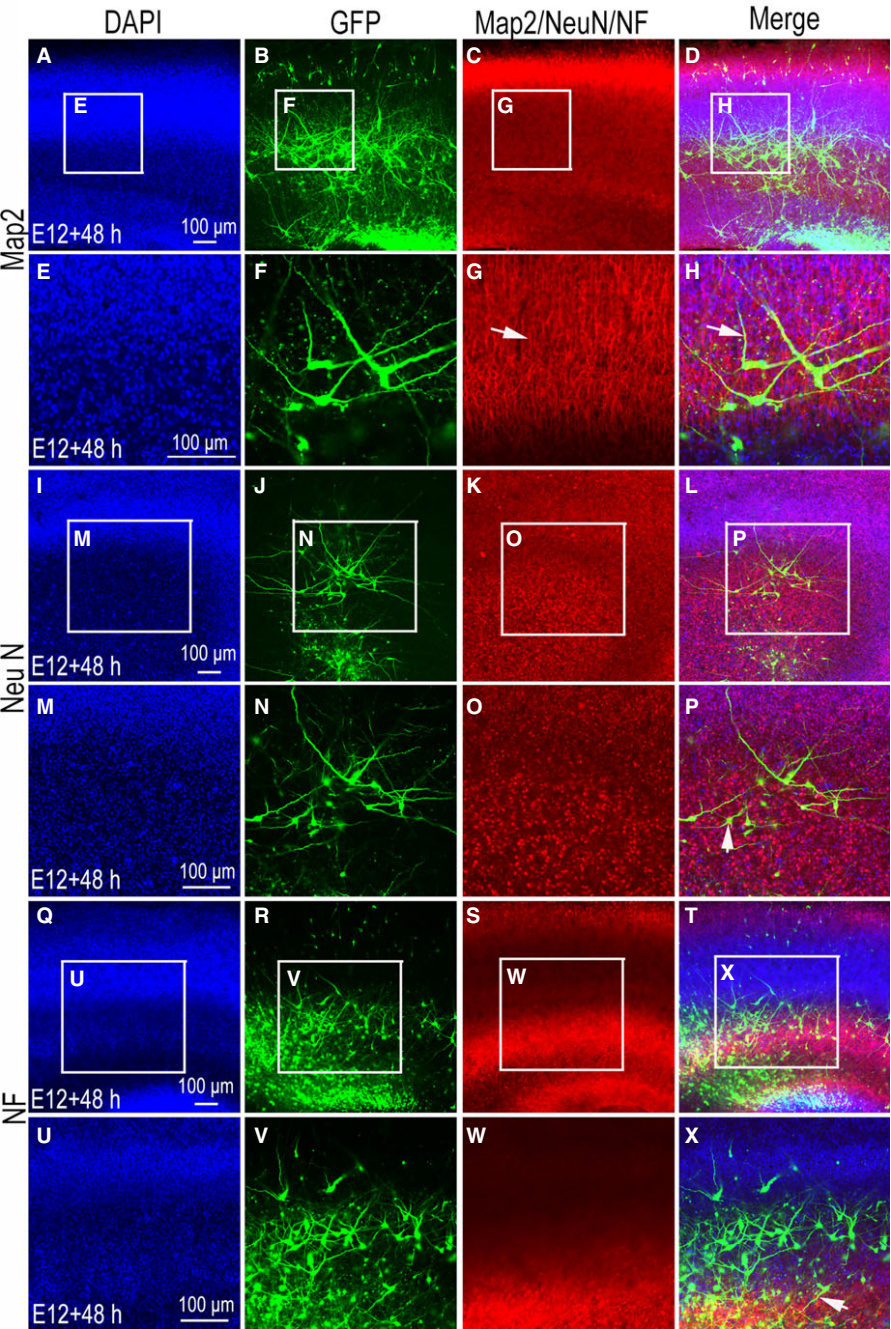
The expression of Map2, NeuN and neurofilament (NF) in cultured chicken optic tectum slice. (A‐X) were imaged using a confocal microscope. (A‐H) the expression of Map2 in pCAGGS‐green fluorescent protein (GFP) positive slices at stage 38 (E12) culture to 48 h; 4ʹ,6‐diamidino‐2‐phenylindole (DAPI) nuclear staining (A, higher magnification areas in E, blue), GFP expression (B, higher magnification areas in F, green), Map2 expression (C, higher magnification areas in G, red), and the merged image (D, higher magnification areas in H) are shown. (I‐P) the expression of NeuN in pCAGGS‐GFP positive slices at stage 38 (E12) cultured for 48 h; DAPI nuclear staining (I, higher magnification areas in M, blue), GFP expression (J, higher magnification areas in N, green), NeuN expression (K, higher magnification areas in O, red), and the merged image (L, higher magnification areas in P) are shown. (Q‐X) the expression of NF in pCAGGS‐GFP positive slices at stage 38 (E12) culture to 48 h; DAPI nuclear staining (Q, higher magnification areas in U, blue), GFP expression (R, higher magnification areas in V, green), NF expression (S, higher magnification areas in W, red) and the merged image (T, higher magnification areas in X) are shown. Scale bars, 100 µm in (A,E,I,M,Q,U) for (A‐X) respectively

In slice culture, the most important observations are of the morphological structure and changes of neurons. Therefore, we performed immunofluorescence detection of three neuronal markers, microtubule‐associated protein‐2 (Map2), NeuN and NF, in cultured slices for 48 hours. These results show that the nucleus of DAPI‐labelled cells has clear contours (Figure 9A,E,I,M,Q,U), GFP‐positive neurons have distinct processes (Figure 9B,F,J,N,R,V), Map2 is strongly positive across the entirety of tissue slices (Figure 9C,G), and, at further magnifications, we can see that GFP expression partially overlaps with that of Map2 (Figure 9D,H; arrow shows GFP expression overlaps with Map2). These results show that NeuN is mainly expressed in the nucleus of neurons (Figure 9K‐O) and coincides with GFP‐positive cells (Figure 9L,P). Except for GFP‐positive cells, there is a large number of NeuN‐positive cells in cultured tissue slices, suggesting that the transfected cells form only a part of them. The results also show that the expression of NF differs across layers (Figure 9Q‐T). While the expression of NF overlaps with some of that of GFP, some GFP‐positive neurons show no expression of NF (Figure 9U‐X; X arrow shows NF overlapped with GFP).

### 3.8 Comparison of IBa1 and GFAP expression in vivo and in cultured slice of mouse cerebral cortex

**Figure 10:**
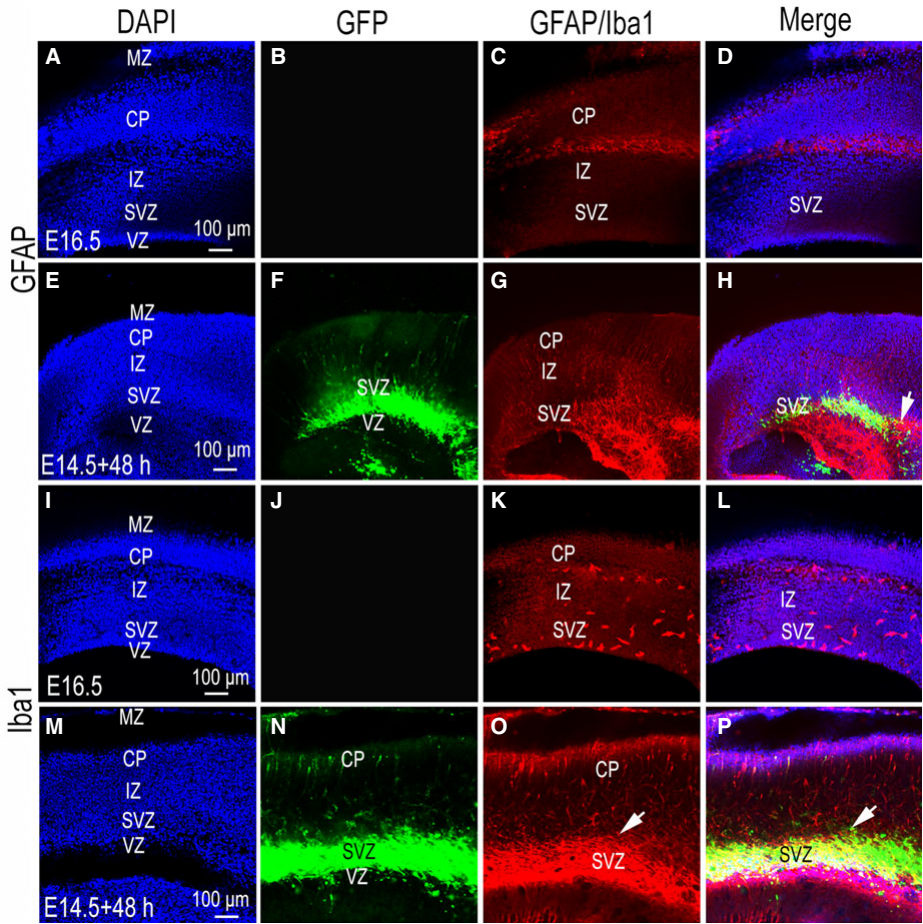
Comparison of GFAP and Iba1 expression in vivo and in culture slices of mouse cerebral cortex. (A‐P) were imaged using a confocal microscope. (A‐D) GFAP expression result in an in vivo slice of mouse cerebral cortex. (A) 4ʹ,6‐diamidino‐2‐phenylindole (DAPI) nuclear staining; (B) green fluorescent protein (GFP) expression, (C) GFAP expression; (A), (B) and (C) merged images are shown (D). (E‐H) culture slice of mouse cerebral cortex GFAP expression result. (E) DAPI nuclear staining; (F) GFP expression, (G) GFAP expression; (E), (F) and (G) merged images are shown (H). Arrow in (H) show GFP and GFAP double‐positive cells. (I‐L) in vivo slice of mouse cerebral cortex Iba1 expression result. (I) DAPI nuclear staining; (J) GFP expression, (K) GFAP expression; (I), (J) and (K) merged images are shown (L). (M‐P) results of Iba1 expression in culture slices of mouse cerebral cortex. (M) DAPI nuclear staining; (N) GFP expression, (O) Iba1 expression; (M), (N) and (O) merged images are shown (P). Arrow in (P) show GFP and Iba1 double‐positive cells. Abbreviations: MZ, Marginal zone; CP, Cortical plate; IZ, Intermediate zone; SVZ, subventricular zone; VZ, ventricular zone. Scale bars, 100 µm in (A,E,I,M) for (A‐P) respectively

In the process of tissue slice culture, we were able to fully characterize various states of neuronal growth through the detection of key neuronal markers. At the same time, we were able to detect glial cells, including microglia and astrocytes. In this experiment, the mouse embryos of E14.5 were obtained from the uterus. In vitro, the brain cortex was transfected with GFP plasmids by in ovo electroporation. Following electroporation, sections were immediately cut and cultured. We analysed and compared mouse brain sections at embryonic development stages up to E16.5 with those of mouse brain slices, labelled with anti‐GFAP and anti‐Iba1, cultured at E14.5 for 48 hours. These results showed that few GFAP‐positive cells were seen in the cerebral cortex of mice developed in vivo, especially in the subventricular zone (Figure 10A‐D), while a large number of GFAP‐positive cells were found in cultured tissue slices in vitro, both in the subventricular zone and other layers (Figure 10E‐H). GFP‐positive cells also expressed GFAP (Figure 10H, arrow show), indicating that astrocytes were also transfected in addition to neurons. The results of Iba1 expression were similar to those of GFAP expression. The expression of Iba1 was low or absent in the control group (Figure 10I‐L), but high in the cultured brain slices, especially in the subventricular zone (Figure 10M‐P). Similarly, Iba1 was expressed in GFP‐positive labelled cells (Figure 10P; arrow show), suggesting that microglia were also transfected.

## 4 DISCUSSION

During CNS development, ectopic expression of genes can lead to structural and functional abnormalities. The chicken embryo is widely used as a model to study gene function during CNS development. With the development of in ovo electroporation technology, it is possible to manipulate and study gene function in the CNS of the chicken embryo. In the CNS, neuronal migration, axonal projection, neuronal path finding, and neural circuit and network formation are recent research topics. Here, we established a method combining in ovo electroporation and the culture of organotypic tissue slices to study exogenous gene functions in the CNS during chicken embryo development. Some of these methods have been established for the CNS tissue slice culture during chicken embryo development.^18^, ^19^ Compared with the established methods, we have combined in ovo electroporation with tissue slice culture to study the function of exogenous genes in the CNS during chicken embryo development. At the same time, we succeeded in performing in ovo electroporation in the spinal cord and the optic tectum. In ovo electroporation was used in the spinal cord or the optic tectum prior to slice culture. The embryo was then allowed to develop to a specific stage, and tissue slices were then sectioned for slice culture. The morphology and structure of neurons can be observed in the cultured slice. In the development of chicken embryos, based on in ovo electroporation, both the spinal cord and optic tectum have their own advantages and disadvantages as research sites as research site. Compared with the optic tectum, the spinal cord in ovo electroporation is well adapted to early embryonic stages (stage 17‐24). It is highly efficient, has high embryonic survival rates and is easy to perform. It was mainly used for contexts such as but not limited to spinal cord commissural axon projection and axon formation. In contrast, the optic tectum in ovo electroporation is suitable for late embryonic stages (stage 24‐28). Achieving successful transfection is difficult and embryo survival rates are low. It was mainly used to study certain effects such as but not limited to, abnormal gene expression on neuronal migration, axonal pathfinding and laminar structure formation. Differences between the spinal cord and optic tectum mean that the methods of in ovo electroporation need to also be different, the main differences being electrode and electro parameters. There are also some differences in later stages of slice tissue culture highlighted in the methods section.

We have found that the morphological structure of the CNS is compromised during the process of serum‐free medium culturing. We further found that the neurons in the spinal cord lose their regular migration patterns and that the direction of commissural axons is changed, in addition the optic tectum losing its distinctive layered structure. This result was as predicted since, considering that cells are living and growing in culture medium, this process causes the organically generated regulatory function of various factors in the organism to be lost. In this experiment, we used serum‐free medium. Despite being in tissue slices, neurons that survive in the medium differ from those of the body as many functions are not completely reflective of the body's natural conditions. This is also a problem that needs to be taken into consideration in slice culture experiments. There are differences between slice cultures in vitro and sections in vivo are not only in terms of gross morphology, but also in terms of fine structure, such as the length of dendrites, spines on dendrites number and axons number.^24^ In this experiment, the histological structure and the length of neuronal axons cultured in serum‐free medium and 25% horse serum medium were compared and analysed. Results showed that the culture medium containing 25% horse serum could better maintain the morphological structure of tissues, and neuronal axons were significantly longer than those cultured in serum‐free medium. In conclusion, neurobasal medium appears suitable for neuronal survival, but lacks the nutrients needed for the growth of non‐neuronal cells. However, adding horse serum is more conducive to the maintenance of tissue morphology and to the survival of neurons in tissue slice culture.

These experimental results show that the process of CNS slice culture is most suitable for single neuron studies. The structure of a complete single neuron can be observed from slice culture. Even though tissue slices lose their original morphological structure during culture, neurons remain in the tissue. Therefore, we are able to observe neuronal migration in the tissue slice and axonal path finding during culture. Though conditions are not exactly reflective of those of the living body, slice culture can still adequately mimic the dynamic changes of neurons in the living body. We were able to successfully observe the complete structure of single neurons. In this experiment, we compared the slice‐cultured tissue with sectioned slices. We found that no complete single neuron could be obtained from sectioned slices, but the complete structure of single neurons could be obtained from slice‐cultured tissue. In addition, prior to slice culture, we performed in ovo electroporation in the spinal cord or optic tectum to achieve the ectopic expression of exogenous genes in neurons. Using slice culture, we were able to analyse the effects of exogenous gene expression on neurons at the single cell level. Because the observed neurons were pre‐transfected with GFP reporter gene, the dynamic changes of GFP‐positive neurons were able to be observed by time‐lapse imaging. We had previously established methods of in ovo electroporation and neuronal culture for gene function analysis of embryogenesis in the chicken optic tectum in earlier studies.^25^ Comparing slices, cultured neurons, and isolated neurons, axons extending from the isolated neurons were found to be quite variable in location, while axonal extensions of neurons in slice culture tissue were more restrained and regular. Compared with the isolation and culture of neurons, tissue slice culture does not require enzymatic digestion, reduces neuronal damage produces results that are more reflective of in vivo conditions. This method provides an effective way to study axon formation and migration of single neurons in vitro.

In this experiment, we compared the effects of GFP expression on cell apoptosis and proliferation in sectioned tissue and slice culture tissue. Results showed that the expression of GFP was neither related to apoptosis nor to cell proliferation. However, a large number of proliferating cells were seen in the tissue culture, especially in the grey matter area. This is a new finding, which warrants further study. In this experiment, neuronal markers in cultured tissue slices were also detected. MAP‐2 belongs to the microtubule‐associated protein family and proteins of this family are thought to participate in microtubule assembly, an essential step in neuritogenesis. MAP‐2 isoforms are found predominately in neurons^26^ and MAP‐2's principal functions are to reduce the critical concentration of tubulin required to polymerize microtubules and to maintain neuronal morphology by regulating microtubule spacing.^27^, ^28^ NeuN is a marker of mature neurons and is expressed in the nucleus. NF is a neurofilament protein whose expression is enhanced in response to inflammation. The detection of neuronal markers also fully demonstrated that neurons have developed into mature neurons with appropriate neuronal functions. They display typical axonal and dendritic structures. At the same time, the axons in serum medium containing 25% horse are significantly longer than those in serum‐free medium.

The results of GFAP and Iba1 showed a significant difference between in vivo and in vitro‐cultured brain slices. The number of GFAP‐ and Iba1‐positive cells increased significantly in cultured‐brain slices. GFAP is considered a marker for astrocytes and Iba1 a marker for microglia. Increased GFAP gene expression is a common feature of CNS injury and is usually used as a marker of nerve injury.^29^ Microglia, the innate immune cells of the CNS, constantly survey CNS parenchyma for pathogens and cellular stress signals.^30^ Many studies have shown that the expression of GFAP will change over the course of organotypic slice culture.^31^, ^32^ The changes in GFAP and Iba1 expression levels reflect differences between the brain slice culture process and in vivo conditions. However, they are also due to damage induced in the process of tissue sectioning. More research is needed to elucidate the differences between organotypic slice culture and in vivo conditions.

## 5 CONCLUSION

We provide a method that combines in ovo electroporation and slice culture to study gene function in chicken CNS during embryonic development. The comparison of serum‐free medium with in vivo and 25% horse serum medium shows that tissue culture and in vivo conditions differ. Adding horse serum is more conducive to the maintenance of tissue structure, and this method is suitable for the study of single neuronal dynamics.

## CONFLICT OF INTEREST

The authors declare that they have no known conflicts of interest associated with this publication.

## AUTHORS’ CONTRIBUTIONS

Juntang Lin conceived and designed the experiments. Ciqing Yang, Xiaoying Li, Shuanqing Li, Xuejun Chai, Lihong Guan, Liang Qiao, Han Li performed the experiments. Ciqing Yang analysed the data and wrote the article..

## References

[1] Gähwiler BH . Organotypic monolayer cultures of nervous tissue. J Neurosci Methods. 1981;4:329‐342. 7033675 10.1016/0165-0270(81)90003-0

[2] Gähwiler BH . Morphological differentiation of nerve cells in thin organotypic cultures derived from rat hippocampus and cerebellum. Proc R Soc Lond B Biol Sci. 1981b;211:287‐290. 6111800 10.1098/rspb.1981.0007

[3] Gähwiler BH . Organotypic cultures of neural tissue. Trends Neurosci. 1988;11:484‐489. 2469173 10.1016/0166-2236(88)90007-0

[4] Stoppini L , Buchs PA , Muller D . A simple method for organotypic cultures of nervous tissue. J Neurosci Methods. 1991;37:173‐182. 1715499 10.1016/0165-0270(91)90128-m

[5] Radic T , Jungenitz T , Singer M , et al. Time‐lapse imaging reveals highly dynamic structural maturation of postnatally born dentate granule cells in organotypic entorhino‐hippocampal slice cultures. Sci Rep. 2017;7:43724. 28256620 10.1038/srep43724 PMC5335612

[6] Kim UJ , Won R , Lee KH . Neuroprotective effects of okadaic acid following oxidative injury in organotypic hippocampal slice culture. Brain Res. 2015;1618:241‐248. 26067888 10.1016/j.brainres.2015.05.039

[7] Guldimann C , Lejeune B , Hofer S , et al. Ruminant organotypic brain‐slice cultures as a model for the investigation of CNS listeriosis. Int J Exp Pathol. 2012;93:259‐268. 22804762 10.1111/j.1365-2613.2012.00821.x PMC3444982

[8] Sherafat A , Hill RA , Nishiyama A . Organotypic slice cultures to study oligodendrocyte proliferation, fate, and myelination. Methods Mol Biol. 2018;1791:145‐156. 30006707 10.1007/978-1-4939-7862-5_11 PMC6372239

[9] Mendes ND , Fernandes A , Almeida GM , et al. Free‐floating adult human brain‐derived slice cultures as a model to study the neuronal impact of Alzheimer's disease‐associated Aβ oligomers. J Neurosci Methods. 2018;307:203‐209. 29859877 10.1016/j.jneumeth.2018.05.021

[10] Mewes A , Franke H , Singer D . Organotypic brain slice cultures of adult transgenic P301S mice–a model for tauopathy studies. PLoS ONE. 2012;7:e45017. 22984603 10.1371/journal.pone.0045017 PMC3439393

[11] Harwell CS , Coleman MP . Synaptophysin depletion and intraneuronal Aβ in organotypic hippocampal slice cultures from huAPP transgenic mice. Mol Neurodegener. 2016;11:44. 27287430 10.1186/s13024-016-0110-7 PMC4903008

[12] Minami N , Maeda Y , Shibao S , et al. Organotypic brain explant culture as a drug evaluation system for malignant brain tumors. Cancer Med. 2017;6:2635‐2645. 28980419 10.1002/cam4.1174 PMC5673912

[13] Lee NM , Chae SA , Lee HJ . Effects of neural stem cell media on hypoxic injury in rat hippocampal slice cultures. Brain Res. 2017;1677:20‐25. 28941572 10.1016/j.brainres.2017.09.018

[14] Humpel C . Organotypic brain slice cultures: a review. Neuroscience. 2015;305:86‐98. 26254240 10.1016/j.neuroscience.2015.07.086 PMC4699268

[15] Jang S , Kim H , Kim HJ , et al. Long‐term culture of organotypic hippocampal slice from old 3xTg‐AD mouse: an ex vivo model of Alzheimer's disease. Psychiatry Investig. 2018;15:205‐213. 10.30773/pi.2017.04.02 PMC5900409 29475217

[16] McCaughey‐Chapman A , Connor B . Rat brain sagittal organotypic slice cultures as an ex vivo dopamine cell loss system. J Neurosci Methods. 2017;277:83‐87. 28012853 10.1016/j.jneumeth.2016.12.012

[17] Kleczkowska P , Kawalec M , Bujalska‐Zadrozny M , et al. Effects of the hybridization of opioid and neurotensin pharmacophores on cell survival in rat organotypic hippocampal slice cultures. Neurotox Res. 2015;28:352‐360. 26286504 10.1007/s12640-015-9553-9

[18] Tubby KC , Norval D , Price SR . Chicken embryo spinal cord slice culture protocol. J Vis Exp. 2013;73:1‐5. 10.3791/50295 PMC3640431 23568251

[19] Das RM , Wilcock AC , Swedlow JR , et al. High‐resolution live imaging of cell behavior in the developing neuroepithelium. J Vis Exp. 2012;62:1‐4. 10.3791/3920 PMC3466664 22525126

[20] Impey S , Mark M , Villacres EC , et al. Induction of CRE‐mediated gene expression by stimuli that generate long‐lasting LTP in area CA1 of the hippocampus. Neuron. 1996;16:973‐982. 8630255 10.1016/s0896-6273(00)80120-8

[21] Hamburger V , Hamilton HL . A series of normal stages in the development of the chick embryo. 1951. Dev Dyn. 1992; 195: 231‐272. 1304821 10.1002/aja.1001950404

[22] Yang C , Li X , Wang C , et al. N‐cadherin regulates beta‐catenin signal and its misexpression perturbs commissural axon projection in the developing chicken spinal cord. J Mol Histol. 2016;47:541‐554. 27650519 10.1007/s10735-016-9698-8

[23] Yang C , Li X , Li Q , et al. Sonic hedgehog regulation of the neural precursor cell fate during chicken optic tectum development. J Mol Neurosci. 2018;64:287‐299. 29285739 10.1007/s12031-017-1019-5

[24] Holopainen IE . Organotypic hippocampal slice cultures: a model system to study basic cellular and molecular mechanisms of neuronal cell death, neuroprotection, and synaptic plasticity. Neurochem Res. 2005;30:1521‐1528. 16362771 10.1007/s11064-005-8829-5

[25] Yang C , Li X , Li Q , et al. Combined use of in ovo electroporation and cultured neurons for gene function analysis of embryogenesis in the chicken optic tectum. NeuroReport. 2017;28:1180‐1185. 28953094 10.1097/WNR.0000000000000903

[26] Tucker RP . The roles of microtubule‐associated proteins in brain morphogenesis: a review. Brain Res Brain Res Rev. 1990;15:101‐120. 2282447 10.1016/0165-0173(90)90013-e

[27] Caceres A , Mautino J , Kosik KS . Suppression of MAP2 in cultured cerebellar macroneurons inhibits minor neurite formation. Neuron. 1992;9:607‐618. 1389180 10.1016/0896-6273(92)90025-9

[28] Kalcheva N , Albala J , O'Guin K , et al. Genomic structure of human microtubule‐associated protein 2 (MAP‐2) and characterization of additional MAP‐2 isoforms. Proc Natl Acad Sci U S A. 1995;92:10894‐10898. 7479905 10.1073/pnas.92.24.10894 PMC40537

[29] Brenner M , Messing A , Olsen ML . AP‐1 and the injury response of the GFAP gene. J Neurosci Res. 2018 10.1002/jnr.24338 PMC6289842 30345544

[30] Hanisch UK , Kettenmann H . Microglia: active sensor and versatile effector cells in the normal and pathologic brain. Nat Neurosci. 2007;10:1387‐1394. 17965659 10.1038/nn1997

[31] Yokose J , Ishizuka T , Yoshida T , et al. Lineage analysis of newly generated neurons in organotypic culture of rat hippocampus. Neurosci Res. 2011;69(3):223‐233. 21145363 10.1016/j.neures.2010.11.010

[32] Miller AP , Shah AS , Aperi BV , et al. Acute death of astrocytes in blast‐exposed rat organotypic hippocampal slice cultures. PLoS ONE. 2017;12(3):e0173167. 28264063 10.1371/journal.pone.0173167 PMC5338800

